# Activated spleen tyrosine kinase promotes malignant progression of oral squamous cell carcinoma via mTOR/S6 signaling pathway in an ERK1/2-independent manner

**DOI:** 10.18632/oncotarget.19911

**Published:** 2017-08-03

**Authors:** Pan Gao, Xianghe Qiao, Haibin Sun, Yi Huang, Jie Lin, Longjiang Li, Xiaoyi Wang, Chunjie Li

**Affiliations:** ^1^ State Key Laboratory of Oral Diseases, West China Hospital of Stomatology, Sichuan University, Chengdu 610041, China; ^2^ Department of Head and Neck Oncology, West China Hospital of Stomatology, Sichuan University, Chengdu 610041, China; ^3^ Department of Oral and Maxillofacial Surgery, Sichuan Provincial People's Hospital, Chengdu 610072, China; ^4^ Department of Dental Anesthesiology, West China Hospital of Stomatology, Sichuan University, Chengdu 610041, China

**Keywords:** SYK, OSCC, piceatannol, ERK1/2, mTOR

## Abstract

Spleen tyrosine kinase (SYK), a non-receptor cytoplasmic tyrosine enzyme, is well known for its ability in certain pathways through immune receptors. Recently, *SYK* role in cancer has been widely studied. *SYK* plays a dual role as a tumor suppressor and tumor promoter. Nevertheless, its role in oral squamous cell carcinoma (OSCC) has not been fully investigated. In the current study, samples from OSCC tumors and adjacent normal counterparts were collected and *SYK* expression was evaluated by real-time qPCR. *SYK* mRNA expression in tumors was higher than the normal tissues. And high SYK expression was confirmed by immunohistochemistry analysis and closely related to worse overall survival. The expression of SYK mRNA and protein was detected in 2 of 4 OSCC cell lines. *SYK* pharmacological suppression and RNAi-mediated knockdown inhibited proliferation, migration, and invasion of *SYK*-positive cells by reducing phosphorylated ERK1/2 *and* mTOR levels. One inhibitor of MEK, PD98059, also suppressed the same cancer-associated phenotypes of *SYK*-positive cells by decreasing phosphorylated ERK1/2 but increasing phosphorylated mTOR. Piceatannol, one pharmacological inhibitor of SYK, attenuated tumor growth *in vivo*. Overall, our results revealed a novel mechanism triggered by *SYK* to increase OSCC tumoriogenesis and tumor progression.

## INTRODUCTION

Spleen tyrosine kinase (SYK), is a cytoplasmic non-receptor tyrosine kinase containing a dual SRC homology 2 (SH2) domain and a COOH-terminal tyrosine kinase domain [1]. Immunoreceptor tyrosine-based activation motif (ITAM) phosphorylation allows SYK recruitment and activation by ITAM binding to SH2 domains [1]. The well-known role of SYK in immune system is to regulate inflammatory responses through B cell receptors, T cell receptors and Fc receptors [1, 2]. SYK is phosphorylated on multiple tyrosine, and it binds to several proteins, triggering downstream signaling [1, 3, 4] involving a variety of biological processes, including osteoclast development [5], platelet functions [6, 7], and vascular development [7]. *SYK* has been associated with cancer in an increasing number of studies, with a dual function. In cancer of epithelial origin, *SYK* is absent in normal breast tissue, benign breast lesions and less malignant breast cancer cell lines [8, 9]. *SYK* ectopic expression in a *SYK*-negative breast cancer cell line significantly suppresses cell motility and metastatic ability [9]. *SYK* expressed in melanocytes facilitates senescence and is lost in melanoma cells due to DNA methylation-mediated gene silencing [10]. In lymphatic and hematopoietic systems, *SYK* acts as a tumor promoter. Indeed, increased activity or overexpression of *SYK* is associated with worse prognosis of patients with acute myeloid leukemia, chronic lymphocytic leukemia, or T- or B-cell lymphoma [11–15], while *SYK* knockdown or inhibition induces apoptosis both *in vitro* and *in vivo* [15, 16]. A phase 2 clinical trial with entospletinib, a selective SYK inhibitor, revealed clinical improvement in patients with relapsed or refractory chronic lymphocytic leukemia [17]. Enhanced *SYK* expression is also implicated in human prostate cancer and related to malignant progression [18]. Ogane et al. ever found that *SYK* is downregulated in oral cancer cell lines as a result of frequent hypermethylation in its CpG island region. They further determined the *SYK* function as a tumor suppressor gene since *SYK* restoration inhibited cells motility and invasiveness [19]. Their results were contradictory to the results obtained by Luangdilok et al. on squamous cell carcinoma of the head and neck (SCCHN), which confirmed that *SYK* acted as an oncogene, promoting cell motility and SCCHN progression, including oral cancer [20]. Therefore, due to its dual function, it is of utmost importance to elucidate *SYK* mechanisms in OSCC. In the current study, SYK overexpression was associated with some clinicopathological characteristics and overall survival of OSCC patients. Further *in vitro* investigations using OSCC cell lines and *in vivo* studies using a nude mice model indicated that *SYK* functions as an oncogene, revealing a novel therapeutic target for OSCC treatment.

## RESULTS

### *SYK* expression is higher in human OSCC tissues and is associated with lymph node metastasis and overall survival

**Figure 1:**
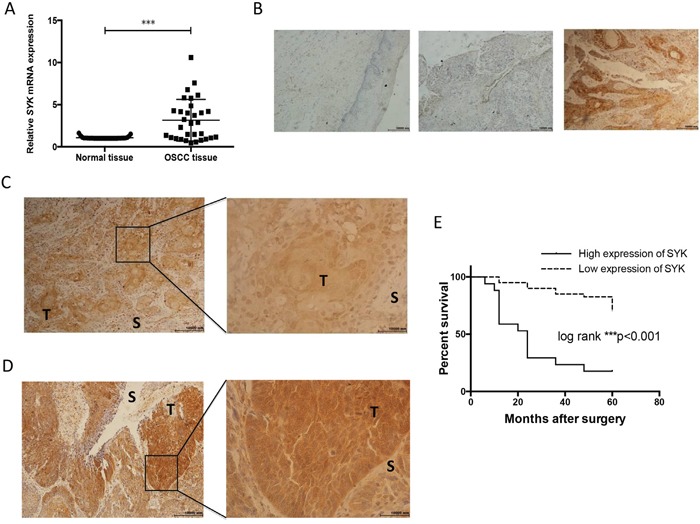
SYK is significantly over-expressed in cancerous tissues and assocaited with overall survival **(A)**: Relative expression of *SYK* in OSCC tissues in comparison with adjacent, histologically normal tissues (n=31). The *SYK* mRNA level in tumor tissues was normalized to normal tissues. **(B)**: Staining of SYK in normal tissues (left). Staining of SYK in OSCC tissues, SYK-negative (middle) and SYK-positive (right) expression in epithelial layers. (magnification, ×100). **(C** and **D)**: Different patterns of SYK expression (C): Cytoplasmic and nuclear staining; (D): Predominantly cytoplasmic staining; T, tumor; S, stroma; magnification, ×100 left and ×400 right) in OSCC tissues. **(E)**: Kaplan-Meier curves for the overall survival rate of p OSCC patients were calculated based on SYK expression (high versus low) determined by IHC. Log-rank test was used to compare the significance of the two curves.

*SYK* expression was measured using RT-qPCR in 31 OSCC samples and adjacent normal tissues. *SYK* mRNA significantly increased in tumor tissues compared with normal counterparts (mean of differences and SEM: 2.08 ± 0.45) (Figure 1A). *SYK* expression among different patients’ clinicopathological characteristics indicated that high *SYK* expression was correlated with lymph node metastasis (Table 1). However, in this study, *SYK* expression was not associated with gender, age, pathologic stage, T classification, clinical stage and recurrence. A retrospective immunohistochemical analysis in OSCC patients with long follow-ups was performed to further elucidate the relationships between *SYK*, clinicopathological characteristics and overall survival. Staining results from paraffin-embedded normal and OSCC tissues are shown in Figure 1B. Both SYK cytoplasmic and nuclear staining were observed in the most of the positive samples (Figure 1C, 1D). Clinicopathological characteristics and SYK expression (high vs. low) on all 57 patients are shown in Table 2. SYK expression was associated with age and recurrence (Table 2). However, no statistically significant correlation was found between SYK expression and gender, pathologic stage, primary tumors size, lymph node status, and postoperative therapies. Overall survival analysis showed that patients with high SYK expression had a significantly worse survival than those with low SYK expression (log-rank test, *P*<0.001; Figure 1E).

**Table 1:** Relationship between Syk mRNA expression and clinicopathologic characteristics in 31 OSCC tissue samples

Characteristics	n (%)	Relative expression of Syk^a^	P^b^
Gender			
Male	18(58)	1.42 (0.91-5.09)	
Female	13(42)	3.72 (1.93-4.25)	0.3045
Age (y)			
<60	13(42)	1.56 (0.95-5.01)	
≥60	18(58)	2.57 (1.08-4.35)	0.9764
Pathologic stage			
I,II	26(81)	2.57 (1.14-4.42)	
III,IV	5(19)	0.98 (0.59-5.89)	0.5087
T classification^c^			
T_1-2_	19(61)	2.78 (1.47-4.18)	
T_3-4_	8(25)	1.04 (0.62-4.62)	0.0926
Lymph node metastasis			
N0	17(55)	2.36 (0.99-4.20)	
N1	4(13)	0.88 (0.50-1.97)	
N2	10(32)	4.29 (1.54-6.49)	**0.0219**
Clinical stage^c^			
I,II	12(39)	2.80 (1.14-4.14)	
III,IV	15(48)	1.56 (0.98-4.85)	0.6572
Recurrence			
No	27(87)	2.30 (1.06-4.18)	
Yes	4(13)	4.25 (1.74-5.67)	0.3414

^a^Median of relative expression (25% percentile-75% Percentile).

^b^Mann-Whitney U test between two groups and Kruskall-Wallis test for 3 groups.

^c^The recurrent patients were not grouped by T classification and Clinical stage.

**Table 2:** Relationship between Syk expression and clinicopathologic characteristics through IHC in 57 patients with OSCC

Characteristics	n (%)	Syk expression		P
		Low	High	
Gender				
Male	32(56)	20	12	
Female	25(44)	20	5	0.1518
Age (y)				
<60	30(53)	26	4	
≥60	27(47)	14	13	**0.0041**
Pathologic stage				
I,II	40(70)	28	12	
III,IV	17(30)	12	5	0.9646
T stage				
T_1-2_	39(68)	29	10	
T_3-4_	18(32)	11	7	0.3095
Lymph node metastasis				
Negative	51(88)	37	13	
Positive	7(12)	3	4	0.0916
Recurrence				
No	53(93)	39	14	
Yes	4(7)	1	3	**0.0405**
Postoperative therapy				
No	29(51)	20	9	
Chemo- or radiotherapy	28(49)	20	8	0.8390

### *SYK* is expressed in some OSCC cell lines

**Figure 2:**
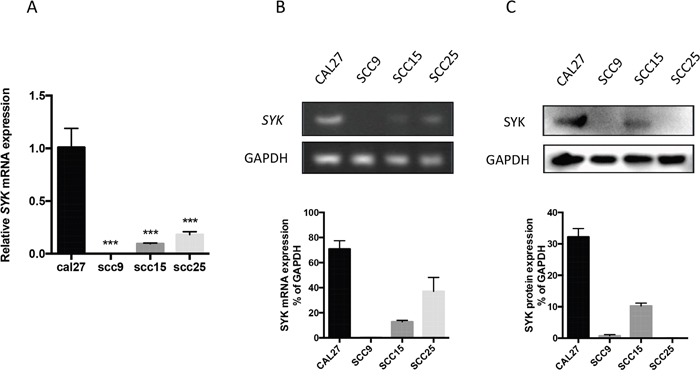
SYK expression in 4 oral cancer cell lines **(A)**: The relative mRNA expression of *SYK* in 4 OSCC cell lines was analyzed by RT-qPCR, GAPDH being a reference gene. **(B)**: RT-PCR and agarose gel electrophoresis were used to verify the expression of *SYK*. **(C)**: OSCC cell lysates were analyzed for SYK expression by Western blot, GAPDH being a loading control. Statistical graphs are shown below. ^***^ p < 0.001.

We assessed SYK expression in 4 OSCC cell lines by PCR (Figure 2A and 2B) and Western blot (Figure 2C). CAL27, SCC15, and SCC25 expressed *SYK*, whereas SCC9 did not (Figure 2B). CAL27 expressed the strongest SYK protein whereas it was weak in SCC15 and no clear expression was found in the other cell lines (Figure 2C).

### SYK inhibition and knockdown reduces proliferation, migration, and invasion of OSCC cells

**Figure 3:**
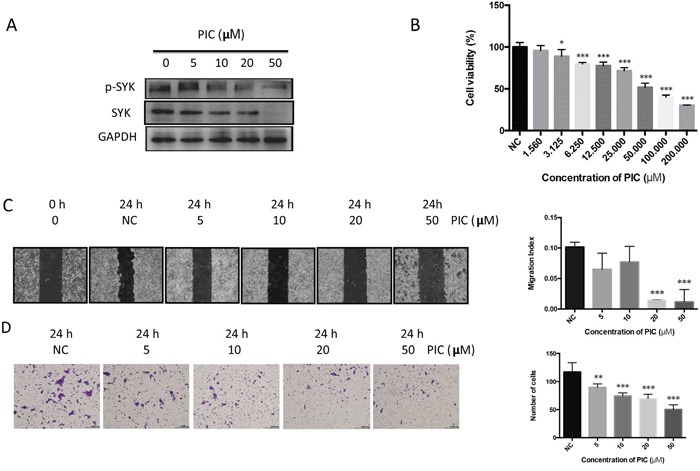
Piceatannol attenuates proliferation, migration, and invasion of OSCC cell line **(A)**: After induced by PIC and vehicle for 24 h, CAL27 cell lysates were harvested and performed Western blot for levels of phosphorylated, total SYK, and GAPDH. **(B)**: CAL27 cells, seeded in 96-well plate for overnight, were stimulated by gradient concentration of PIC (from 1.56 to 200 μM) and the vehicle. Cell viability was detected by CCK8 assay. **(C)**: Representative images (left) were obtained from the wound healing assay induced by PIC. Quantitative analysis of the migration was indicated using migration index (right). **(D)**: Representative images (left) and the quantitative analysis (right) showed the invasion ability of CAL27 cells after induced by PIC using transwell invasion assay. ^**^ p < 0.01; ^***^ p < 0.001.

**Figure 4:**
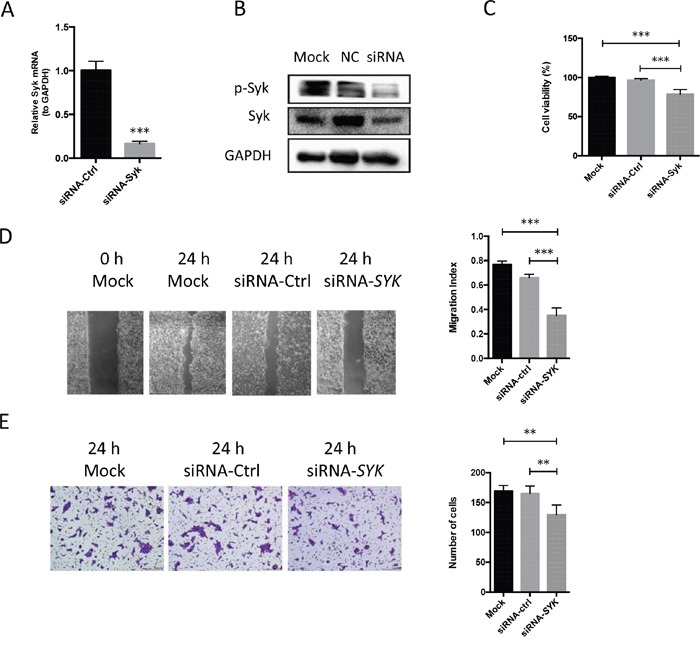
SYK Knockdown in OSCC cell lines inhibits cell proliferation, migration, and invasion **(A)**: *SYK* mRNA expression by RT-qPCR analysis for cells transfected by siRNA-*SYK* (25 nM) and the contrast (siRNA-control) (25 nM) for 24 h. **(B)**: After transfected by siRNA for 48 h, cell lysates were obtained and evaluated for expression of phosphorylated, total SYK, and GAPDH by Western blot. **(C)**: CAL27 cells were seeded in 96-well plate and transfected by siRNA-*SYK* and siRNA-control. CCK8 assay was used to detect cell viability. **(D)**: Representative images (left) were obtained from the wound healing assay transfected by siRNA-*SYK* and siRNA-ctrl. Quantitative analysis of the migration was indicated using migration index (right). **(E)**: Representative images (left) and the quantitative analysis (right) indicated the invasion ability of CAL27 cells after transfected by siRNA-*SYK* and siRNA-control using transwell invasion assay. ^**^ p < 0.01; ^***^ p < 0.001.

Piceatannol, a natural diphenylethene and a hydroxystilbene derivative of resveratrol, suppresses SYK biological activity, thus it is considered as a selective SYK inhibitor [21]. Western blot analysis demonstrated that piceatannol remarkably reduced the expression of phosphorylated SYK at a concentration of 10 μM and enhanced its inhibition at 20 and 50 μM (Figure 3A). Besides, 50-μM piceatannol dramatically inhibited total SYK (Figure 3A). CCK8 assay was performed to investigate cell viability of CAL27 cells treated by piceatannol (Figure 3B). Piceatannol IC50 on CAL27 cells, calculated by GraphPad Prism 6.0, was 44.82 μM. Thus, we considered a concentration range based on both the IC50 we obtained and previous studies using human cancer cells for our further investigation. Piceatannol also inhibited the migration and invasion of CAL27 cells evaluated by the wound healing assay (Figure 3C) and Transwell invasion assay (Figure 3D). Moreover, *SYK* siRNA was used to silence *SYK*. *SYK* silencing significantly decreased *SYK* mRNA level (Figure 4A) and SYK protein level, both the phosphorylated and total SYK (Figure 4B), in CAL27 cells. Similarly, *SYK* knockdown dramatically attenuated proliferation (Figure 4C), migration (Figure 4D), and invasion (Figure 4E) of CAL27 cells compared with Mock and siRNA-Control.

### ERK1/2 and mTOR signaling pathway are associated with SYK activity in OSCC cells

**Figure 5:**
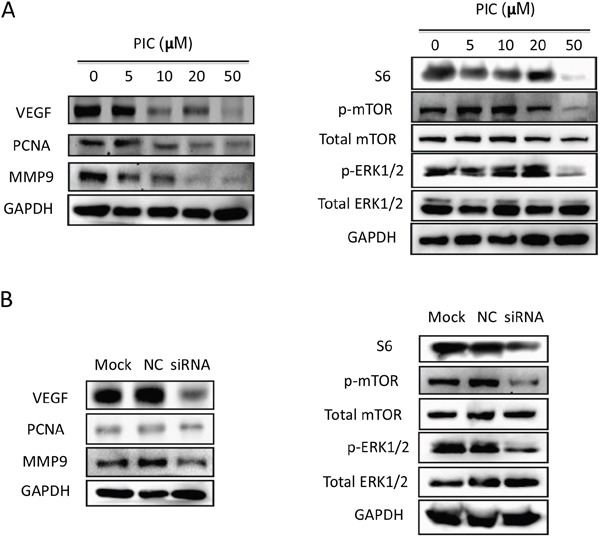
SYK inhibition and knockdown blocks ERK1/2 and mTOR/S6 signaling pathway **(A)**: The cell lysates of CAL27 cells induced by gradient concentration of PIC for 24 h were subjected for expression of VEGF, PCNA, MMP9, GAPDH (left) and phosphorylated, total ERK1/2, mTOR, S6 and GAPDH (right). **(B)**: The cell lysates of CAL27 cells transfected by siRNA-*SYK* and siRNA-control (25 nM) for 48 h were detected by Western blot for expression of VEGF, PCNA, MMP9, GAPDH (left) and phosphorylated, total ERK1/2, mTOR (right).

Western blot analysis was performed to further investigate the intracellular signaling pathways. Both piceatannol and *SYK* knockdown reduced the expression of vascular endothelial growth factor (VEGF), proliferating cell nuclear antigen (PCNA), and metal matrix proteinase (MMP9) (Figure 5A and 5B, left). Phosphorylated extracellular regulated protein kinases 1/2 (ERK1/2), phosphorylated mammalian target of rapamycin (mTOR) and ribosomal protein S6 were notably decreased by piceatannol treatment at 50 μM (Figure 5A, right) and *SYK* knockdown at 25 nM (Figure 5B, right).

### SYK activates mTOR/S6 signaling pathway in an ERK1/2-independent manner

**Figure 6:**
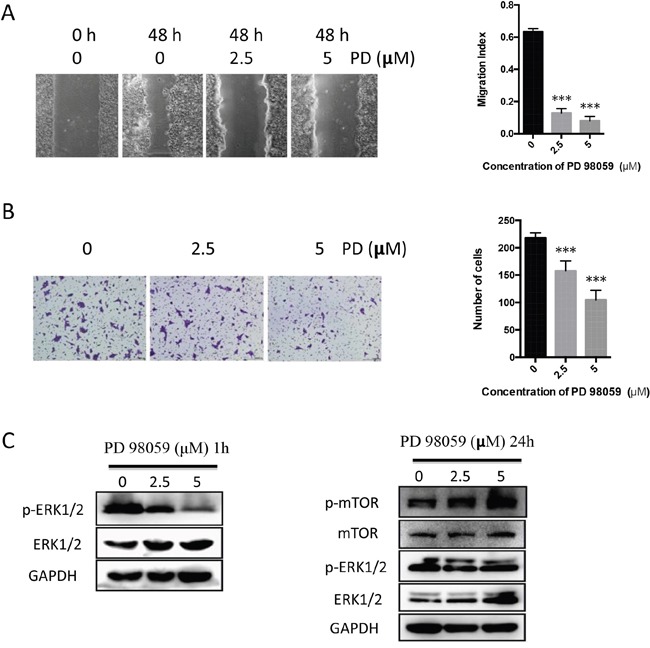
PD98059 attenuates migration and invasion of CAL27 cells whereas up-regulates p-mTOR **(A)**: Representative images (left) indicated the migration of cells induced by PD98059 (2.5, 5 μM) and the vehicle for 48 h. Migration index was used to present the statistic result (right). **(B)**: Representative images (left) and histogram of invasion analysis (right) from transwell assay indicated invasion ability of cells induced by PD98059 and the vehicle for 24 h. **(C)**: Cell lysates from PD98059-treated cells for 1 h (left) and 24 h (right) were analyzed the levels of phosphorylated, total ERK1/2, mTOR and GAPDH using Western blot.

PD98059, a potent inhibitor of mitogen-activated protein kinase (MEK), was used to further study the association between ERK1/2 and mTOR involved in the SYK signaling pathway. PD98059 at 2.5 or 5 μM prominently decreased cell migration (Figure 6A) and invasion (Figure 6B). Phosphorylated ERK1/2 decrease in PD98059-treated (2.5, 5 μM) cells was observed at 1 h (Figure 6C, left), whereas no changes were observed at 24 h (Figure 6C, right) since ERK1/2 phosphorylation is a rapid process. Conversely, increased p-mTOR level was observed in PD98059-treated (2.5, 5 μM) cells at 24 h (Figure 6C, right).

### Piceatannol attenuates tumor growth in OSCC xenograft mice model

**Figure 7:**
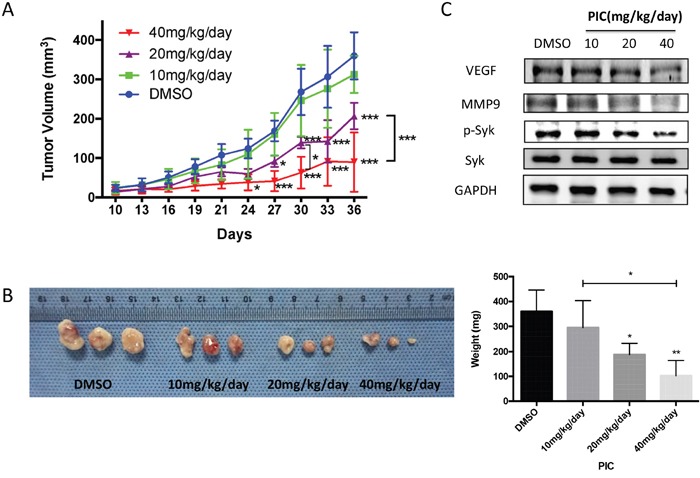
Piceatannol suppresses tumor growth in CAL27 xenograft mice *in vivo* **(A)**: Graph represented growth curves of the subcutaneously injected tumors. **(B)**: Macroscopic appearance of the dissected tumors (left) from mice on the day of sacrifice and tumor weight curves (right). **(C)**: The protein expression of VEGF, MMP9, total and phosphorylated SYK in tumor tissues of the nude mice, administrated by gradient concentration of PIC, was evaluated by western blot. ^*^ p < 0.05; ^**^ p < 0.01; ^***^ p < 0.001.

In order to confirm the role of piceatannol targeting to *SYK* as a tumor suppressor *in vitro*, we established an *in vivo* mice model to examine the effect of piceatannol on OSCC tumor growth. During 36 days observation, we found that piceatannol significantly reduced tumor growth from day 27 onward at a dosage of 20 mg/kg/day, and from day 24 onward at a dosage of 40 mg/kg/day compared with DMSO vehicle (Figure 7A). And the differences were dose-dependently at 30 days and 36 days (Figure 7A). As shown in Figure 7B, the average tumor weight, measured at the end of the experiment, varied in different groups and suggested piceatannol's potently inhibitory effect against tumor growth. Western blot analysis showed that VEGF, MMP9, and phosphorylated SYK protein in tumor tissues of the nude mice decreased with increasing dosage of piceatannol (Figure 7C), which was consistent with the *in vitro* assay.

## DISCUSSION

Oral and maxillofacial malignant tumors incidence is approximately 8 percent of all human cancers in China. Most of the malignant tumors are squamous cell carcinoma (SCC), originated from squamous epithelium, which is the most common cancer form, accounting for approximately 90 percent of all oral cavity carcinomas. For most of the patients, a single surgical treatment is not enough to completely remove the tumor, thus traditional radiotherapy, chemotherapy or chemoradiotherapy is usually recommended. In recent years, the surprising progresses of molecular targeted anti-tumor drugs convince that targeted therapy on oral cancer is promising. Considering the dual function of SYK reported by previous literatures, we sufficiently proved the relevance between SYK and OSCC through RT-qPCR analysis and immunohistochemistry staining. Collectively, high SYK expression in tumor tissues was associated with lymph node metastasis, recurrence and worse overall survival. Our results are contrary to the outcomes on breast cancer, in which *SYK* expression is decreased in patient samples and cells from low grade malignancy to high malignancy [22]. However our findings are consistent with the results of Luangdilok et al. who suggested *SYK* functioned as an oncogene and might be of prognostic value in SCCHN (including oral cancer) through its role in cell migration and invasion [20].

In the current study, the effect of *SYK* inhibition on OSCC was investigated to explore the potential mechanisms. Proliferation, migration and invasion of tumor cells are essential abilities for cancer progression and metastasis. Piceatannol, a selective SYK inhibitor, similar to *SYK* knockdown, suppressed these malignant phenotypes on *SYK*-positive CAL27 cells, suggesting the significant role of *SYK* on oral cancer progression. VEGF, known for its role of stimulating angiogenesis and promoting tumor growth, is produced by several types of cells, including cancer cells [23] and can enhance cell survival, migration and differentiation by a paracrine and autocrine manner in tumor microenvironment (TME) [24]. In our study, VEGF decrease induced by SYK inhibitor and *SYK* silencing indicated that SYK was involved in the regulation of VEGF signaling. Similar observations were found on PCNA, an essential factor for DNA replication, DNA repair, chromatin assembly and epigenetic inheritance [25], and MMP9, involved in degradation of the extracellular matrix (ECM) in both normal physiological and pathological processes [26]. Mallick et al. observed that PCNA is associated with nodal metastasis and disease-free survival, and could be a predictive prognostic factor for oral cancer [27]. ATX-101, a peptide of PCNA-interacting motif induced apoptosis of multiple myeloma cells and increased the anticancer activity of melphalan in the xenograft mice model [28]. Decomposition of ECM by enzymes in the TME is indispensable for tumor progression, including local invasiveness and metastasis. MMP9 level is associated with the invasive ability of tumor cells and metastasis in xenograft animal models [29]. Collectively, VEGF, PCNA and MMP9 may modulate the proliferation, migration, and invasion of oral cancer cells under the regulation of SYK.

Moreover, both ERK1/2 and mTOR/S6 signaling were involved in the suppression of proliferation, migration and invasion of cancer cells induced by SYK. Two classical signaling pathways, PI3K/AKT and MAPK/ERK, regulate mTOR expression [30, 31]. In order to determine whether mTOR/S6 signaling was an ERK1/2-dependent mechanism associated with SYK inhibition, we used a MEK inhibitor, PD98059, which reduced ERK1/2 phosphorylation whereas increased mTOR phosphorylation. Our findings suggested that mTOR/S6 was involved in SYK-inhibited signaling in an ERK1/2-independent manner. Combined, SYK might modulate cell growing, migratory and invasive ability by regulating ERK1/2 (MAPK) and mTOR signaling pathway respectively. These observations are in accordance with a study of Leseux et al. who found that SYK is an upstream regulator of mTOR through PI3K/AKT-dependent pathway in follicular lymphoma (FL) cells, and SYK-mTOR plays a key role in FL survival [32].

Piceatannol, a SYK selective inhibitor, exhibited anticancer abilities by inhibiting proliferation and inducing apoptosis in many different tumors, including leukemia, lymphoma, melanoma, prostate and colon cancers [21, 32–35]. NF-κB and JAK-1/STAT are involved in piceatannol-stimulated signaling pathway [21]. In our study, piceatannol exerted a potent inhibitory effect on OSCC development in tumor xenograft mice *in vivo* and attenuated VEGF and MMP9 expression in tumor tissues.

Collectively, our current study demonstrates that SYK may be of progressive and prognostic value, and also a novel therapeutic target for OSCC.

## MATERIALS AND METHODS

### Clinical OSCC samples and paraffin-embedded tissue sections

Fresh samples were collected from 31 patients who received surgical treatment at West China Hospital of Stomatology, Sichuan University, China. The tumor samples were obtained from the none-necrotic areas of tumors and the adjacent, histologically normal tissues were obtained from the places that were more than 1.5 cm from the tumor margins. All samples were confirmed by pathological analysis. Paraffin-embedded tumor sections were obtained from other 57 patients diagnosed with OSCC with longer follow-up from the Department of pathology, West China Hospital of Stomatology, Sichuan University, China. Our study protocols were approved by the Medical Ethics Committee of West China Hospital of Stomatology, Sichuan University (WCHSIRB-D-2016-049) and patients’ samples were collected after obtaining an informed consent and all investigations have been performed in accordance with the principles embodied in the Declaration of Helsinki.

### Immunohistochemistry

Slides from paraffin-embedded tissue sections were de-waxed in xylene and rehydrated with ethanol in gradient concentration. Endogenous peroxidase was removed using 3% H_2_O_2_ and antigens were retrieved through microwave oven in 10 mM citrate buffer (pH 6.0). Nonspecific binding antigens were blocked by normal goat serum. Slides were incubated with antibody anti-SYK (rabbit monoclonal; 1:500; cell signaling technology) at room temperature for 1 h and then overnight at 4°C. After washing 3 times with PBS, slides were incubated with peroxidase-conjugated streptavidin (Dako, Glostrup, Denmark), and finally with DAB (ZSGB-BIO, Beijing, China). The developing process was stopped until the desired staining density was reached and then slides were counterstained with hematoxylin. Immunoreactive score system was used to evaluate the semi-quantitative SYK expression.

### Cell culture, treatment and reagents

OSCC cell lines (CAL27, SCC9, SCC15, SCC25) were obtained from State Key Laboratory of Oral Diseases, Sichuan University, China. CAL27 was cultured in DMEM-high glucose (HyClone, USA) containing 10% fetal bovine serum (FBS) (Gibco, Grand Island, USA), penicillin (100 U/ml) and streptomycin (100 μg/ml) (HyClone). SCC9, SCC15 and SCC25 were cultured in DMEM/F12 (1:1) (HyClone) supplemented with 10% FBS (Gibco), penicillin (100 U/ml) and streptomycin (100 ug/ml) (HyClone), and 400 ng/ml hydrocortisone (Sangon Biotech, Shanghai, China). All cells were incubated at 37 °C in a humidified incubator with 5% CO_2_. Piceatannol was purchased from Selleck Corp. (Shanghai, China) and PD98059 from Calbiochem Corp. (La Jolla, USA). Both of the compounds were dissolved in DMSO.

### RT-qPCR analysis

Cells at 2 × 10^5^ in 2 ml complete medium were seeded in a 6-well plate. When confluence was reached to 80%, cells were treated as indicated in the figure legends. Total RNA from tissues and cells were isolated using Trizol (Invitrogen) and PureLink^®^ RNA Mini Kit (Thermo Fisher Scientific Inc., USA) respectively. RNA was reversely transcribed to cDNA by using PrimeScriptTM RT reagents kit (Takara, Dalian, China). Real-time quantitative PCR (RT-qPCR) was performed using SYBR^®^ Premix Ex Taq™ II kit (Takara) by an ABI 7300 Real-time PCR system (Applied Biosystems, USA). RT-qPCR results were normalized to the expression of a reference gene, Glyceraldehyde-3-phosphate dehydrogenase (GAPDH), and calculated by 2^-ΔC^_T_ or 2^-ΔΔC^_T_ method [36]. All performances were according to the manufacturers’ instructions. SYK and GAPDH primer sequences were the following, as described by Luangdilok et al.[20]: SYK forward, 5'-ACTTGGTCAGCGGGTGGAAT-3'; SYK; reverse: 5'-GGGTGCAAGTTCTGGCTCAT-3'; GAPDH forward: 5'-GCACCGTCAAGGCTGAGAAC-3'; GAPDH reverse: 5'-GTGGTGAAGACGCCAGTGGA-3’.

### Western blot analysis

Cells at 5 × 10^5^ in 5 ml complete medium were seeded in a T25 flask. When confluence was reached to 80%, cells were treated as indicated in the figure legends. Total cell proteins and mouse tumor tissues were extracted by RIPA buffer (Beyotime, Nantong, China) and the lysates were sonicated on ice discontinuously for 15 seconds. Protein concentration was determined with BCA Protein Assay Kit (Beyotime). Samples, containing equal amounts of protein (20 μg), were subjected to electrophoresis using 10% SDS-PAGE gel (Bio-Rad, USA) and transferred to nitrocellulose membranes (0.2 μm) (Millipore, USA), and probed sequentially with antibodies against p-SYK (Tyr525/526), SYK, MMP9, p-ERK1/2 (Thr202/Tyr204), ERK1/2, p-mTOR (Ser2448), mTOR, S6 (1:1000; Cell Signaling Technology, USA), VEGF, PCNA (1:1000; Santa Cruz Biotechnology, USA), GAPDH (1:1000; Millipore, USA). After incubation with peroxidase-conjugated secondary antibodies 1:2000 (Dako, Denmark), blots were visualized using Immobilon™ Western Chemiluminescent HRP Substrate (Millipore, USA) under Gel Doc XR+ System with Quantity One software (Bio-rad).

### Small interfering RNA treatment

CAL27 cells were transfected with small interfering RNA (siRNA; 25 nM final concentration) using Lipofectamine RNAiMAX (Invitrogen) and Opti-MEM Reduced Serum Media (Gibco) according to the manufacturers’ instructions. SiRNA-SYK and siRNA-Control were designed by GenePharma (Shanghai, China), and their sequences were the following: siRNA-SYK, GAACUGGGCUCUGGUAAUU; siRNA-Ctrl, UUCUCCGAACGUGUCACGU.

### Cell proliferation assay

CCK-8 assay kit (DOJINDO, Kumamoto, Japan) was used to examine cell proliferation. CAL27 cells (1 × 10^4^ cells) in 100 μl medium were seeded in a 96-well plate. Cells were incubated with CCK-8 reagent for 2 h after piceatannol treatment at different concentrations, or after SYK silencing with siRNA-Ctrl or siRNA-SYK, or after PD98059 treatment as indicated in figure legends, and optical density (OD) values were measured at 450 nm.

### Wound healing assay

Cells at 2 × 10^5^ in 1 ml complete medium, were seeded in a 12-well plate. When confluence was reached, a pipette tip was used to scrape a straight line on each monolayer. Medium with or without the above-mentioned compounds was replaced after 3 washes with PBS to remove detached cells. Cells were incubated at 37°C for 24 h (piceatannol), or 48 h (siRNA and PD98059). Wound widths at five different positions were measured at the beginning and the end of the assay. Results were expressed by the migration index, which is the distance covered by migrated cells in the presence of piceatannol, siRNA, or PD98059 relative to the distance covered by migrated cells in the controls. Experiments were carried out in triplicate independently.

### Cell invasion assay

Cell invasion assay was performed in 24-well Transwell system with 8-μm pore size of polycarbonate filters (Corning, USA). 1 × 10^5^ CAL27 cells in 200 serum-free medium containing 0.1% BSA were seeded in the upper chamber coated with Matrigel (BD Biosciences, CA, USA). The lower chamber contained DMEM medium with 10% FBS. After treatment with piceatannol, siRNA, or PD98059 as indicated in the figure legends for 24 h, the non-penetrating cells on the upper surface of the filter were wiped out using cotton swabs. After fixation in 4% paraformaldehyde for 5 min and permeabilization by methanol for 20 min, invasive cells were stained with 0.1% crystal violet for 15 min. Filters were washed 3 times in PBS after each step and fixed on the slide. Five random fields of the filter were selected and photographed using a light microscope. Image J software (NIH, USA) was used to perform the quantitative analysis.

### Piceatannol *in vivo* antitumor assay

Animal experiments were approved by the Ethics Committee of State Key Laboratory of Oral Diseases, Sichuan University (WCCSIRB-D-2016-019). Animal care and handling were performed according with laboratory animal welfare regulations. Male BALB/c nude mice (4-6 weeks old) were purchased from Dashuo Laboratory Animal Technology Co, Ltd. (Chengdu, China). Housed with free access to food and water, mice were acclimated for one week prior the experiment. CAL27 cells (5 × 10^6^) in 100 μl DMEM medium were subcutaneously implanted on the hind flank of each mouse. Tumor size was measured every 3 days using digital caliper and volumes were estimated via the equation V = π/6 (l × w × h). When tumor volumes reached an approximate volume of 30 mm^3^, mice were randomly divided into 4 groups: vehicle (1% DMSO + 30% polyethylene glycol + 1% Tween 80) and piceatannol at different concentrations (10 mg/kg/day, 20 mg/kg/day, 40 mg/kg/day). Vehicle and piceatannol were intraperitoneally administrated, and tumors were harvested on day 36 when ulcers appeared at the tumor surfaces of most mice.

### Statistical analysis

Difference in SYK mRNA level between normal and cancer tissues was analyzed by paired *t*-test. SYK mRNA significance among clinicopathological features was analyzed by non-parametric test (Mann-Whitney U test or Kruskall-Wallis test). Chi-square test was performed to analyze the associations between SYK expression and clinicopathological characteristics. Overall survival curves and median survival times were calculated using the Kaplan-Meier method, and groups compared by the log-rank test. Results are presented as mean ± SD unless otherwise noted. Significance was analyzed by Student's *t*-test (2 groups) or One-way ANOVA (≧3 groups). *P* value less than 0.05 was considered statistically significant. All statistical analyses were performed using GraphPad Prism 6.0 (GraphPad Software Inc., USA).

## Abbreviations

SYK: spleen tyrosine kinase
OSCC: oral squamous cell carcinoma
SCCHN: squamous cell carcinoma of the head and neck
VEGF: vascular endothelial growth factor
PCNA: proliferating cell nuclear antigen
MMP9: metal matrix proteinase 9
ERK1/2: extracellular regulated protein kinases 1/2
m-TOR: mammalian target of rapamycin
